# Can radiomics signatures and machine learning methods reinforce the revived role of ^18^F-NaF in metastatic bone disease?

**DOI:** 10.22038/aojnmb.2025.88516.1640

**Published:** 2026

**Authors:** Mai Amr Elahmadawy, Dina Hosny Gamal El-din, Shaimaa Farouk Abdelhai, Mona H. Ibrahim, Mohamed Ibrahim, Salma Badr

**Affiliations:** 1 Nuclear Medicine unit, National cancer Institute (NCI), Cairo University, Egypt; 2 Radiodiagnosis department, Faculty of Medicine, Cairo University, Egypt; 3 Clinical oncology and Nuclear Medicine department, Faculty of Medicine, Zagazig University, Egypt; 4 Physics department, Faculty of Science, Zagazig University, Egypt; 5 School of information Technology, New Giza University, Egypt; 6 Nuclear medicine department, Children’s Cancer Hospital, Egypt

**Keywords:** NaF PET, Radiomics, Machine learning, Bone metastases A B S T R A C T

## Abstract

**Objective(s):**

To evaluate whether radiomic features extracted from ^18^F-NaF PET/CT scans, analyzed using machine learning (ML) methods, can improve the differentiation between true metastatic bone lesions (TP) and false-positive benign uptake (FP), thereby enhancing the diagnostic utility of ^18^F-NaF PET/CT.

**Methods:**

This retrospective study included 62 patients with known primary malignancies who underwent ^18^F-NaF PET/CT. Lesions were classified as TP or FP based on consensus interpretation including follow-up. Patients were randomly split into training (n=41) and validation (n=21) groups. Radiomic features were extracted from PET images using LIFEx software. Feature selection (ANOVA, RFE) and ML model training (SVM, Random Forest, XGBoost) were performed. Model performance was evaluated using accuracy, specificity, sensitivity, and AUC, initially with a train/validation split and subsequently with 5-fold cross-validation incorporating feature engineering and hyperparameter tuning. Feature importance was assessed using SHAP.

**Results:**

Significant differences in SUV_max_ (p=0.006) and SUV_mean_ (p=0.034) were observed between TP and FP lesions. Initial validation showed XGBoost performed best (AUC=0.78). After optimization and 5-fold cross-validation on the combined dataset (n=62), the tuned XGBoost model achieved the highest performance (Mean Accuracy: 85.7% ±2.9%, Mean AUC: 0.86), outperforming Random Forest (AUC: 0.79) and SVM (AUC: 0.74). SHAP analysis identified SUV_max_, SUV_mean_, Voxel Volume Num, GLRLM RLNU, and Skew.

**Conclusion:**

Radiomics-based machine learning classifiers, particularly XGBoost, demonstrated strong performance in distinguishing true metastatic from false-positive benign lesions on ^18^F-NaF PET/CT. Integrating radiomics and ML can potentially improve the diagnostic accuracy and robustness of ^18^F-NaF PET/CT for assessing bone metastases. Further validation in larger cohorts is warranted.

## Introduction

 Fluorine-18 sodium fluoride (^18^F-NaF) is a positron-emitting radiopharmaceutical initially introduced for bone lesion detection in 1972 by Blau et al. (1). However, its use declined with the rise of Technetium-99m (Tc-99m) based bone scintigraphy, primarily due to the widespread availability of Tc-99m generators and gamma cameras. In recent years, significant advance-ments in positron emission tomography (PET) technology, particularly the development and implementation of hybrid PET/computed tomography (PET/CT) systems offering higher image resolution, have led to a resurgence of interest in ^18^F-NaF PET/CT for detecting bone metastases (2, 3). Despite its high sensitivity, ^18^F-NaF PET/CT can suffer from lower specificity, as uptake can occur in various benign conditions like fractures, degenerative changes, and inflammatory processes, leading to false-positive findings (4). Radiomics involves the high-throughput extraction of quantitative features from medical images, transforming them into mineable data that can potentially uncover underlying pathophysio-logy invisible to the naked eye (5, 6) This approach aims to enhance diagnostic accuracy, improve prognostication, and provide decision support for personalized medicine. Radiomic features typically include first-order statistics (describing voxel intensity distribution), shape-based features, and second- or higher-order textural features that quantify spatial heterogeneity within the region of interest (ROI). The standard radiomics workflow encompasses image acquisition, pre- processing, ROI segmentation, feature extraction and selection, and subsequent analysis using machine learning or deep learning models (7) Machine learning (ML) algorithms are designed to learn patterns from data and make predictions or classifications without explicit programming. When integrated with radiomics, ML models can analyze complex, high-dimensional feature sets to build predictive tools for various clinical applications (8, 9) Previous studies have successfully employed machine learning-based radiomics for tasks such as classifying solitary pulmonary lesions using ^18^F-FDG PET/CT (10) predicting clinical outcomes in prostate cancer (11) and forecasting recurrence in hepatocellular carcinoma based on CT images (12) These applications highlight the potential of combining quantitative imaging features with advanced computational methods to improve clinical decision-making. Given the challenges in differentiating benign from malignant uptake on ^18^F-NaF PET/CT, there is a clinical need for methods that can improve diagnostic specificity. Therefore, this study aims to investigate whether radiomic signatures derived from ^18^F-NaF PET images, analyzed using various machine learning methods, can enhance the diagnostic performance in discriminating true metastatic bone lesions from false-positive findings.

## Methods

***Study Population***

 This retrospective study received approval from the Institutional Research Ethics Committee (IRB number 421/29-Sep-2024), and the requirement for informed consent was waived. We enrolled 62 adult patients with pathologically confirmed primary malignancies who were referred to the Nuclear Medicine unit at the National Cancer Institute between June 2018 and October 2019 for an initial pre-therapy ^18^F-NaF PET/CT assessment. Sodium Fluoride fluorine-18 (^18^F-NaF) was produced at the Children’s Cancer Hospital Egypt (CCHE 57357).

**Figure 1:**
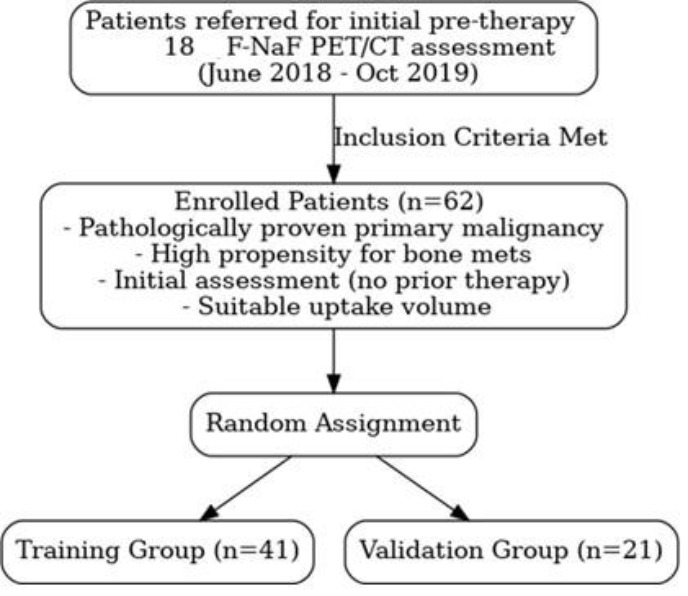
Flowchart illustrating the patient selection process. Patients referred for initial ^18^F-NaF PET/CT were screened, and 62 eligible patients were enrolled and randomly assigned to training (n=41) and validation (n=21) groups

 The inclusion criteria were as follows: (1) Patients aged 18 years or older with pathologically proven primary malignancy and a high clinicolaboratory propensity for bone metastases (e.g., high-risk disease or clinical suspicion). (2) Patients referred for an initial staging assessment before receiving any chemotherapy or radiotherapy. (3) Patients exhibiting focal ^18^F-NaF uptake on PET/CT scans suitable for textural feature extraction (i.e., sufficient volume) (Figure 1).

Clinical information, including age, sex, primary tumor type and pathology, diagnostic methods, imaging findings from other modalities (if available), treatment response, and survival data, was extracted from the institutional medical records.

 The enrolled patients were randomly divided into a training group (n=41) and an independent validation group (n=21) for model development and evaluation, respectively.

^18^ ***F-NaF PET/CT Image Acquisition***

***Patient Preparation***

 Patients were instructed to maintain good hydration by drinking water between the time of radiopharmaceutical injection and imaging session. They were also advised to void their bladder frequently during this interval, including immediately before the scan commenced.

***Acquisition Protocol***

 All ^18^F-NaF PET/CT scans were performed on a Discovery PET/CT scanner (GE Medical Systems, USA). Imaging commenced approximately 60 minutes after the intravenous administration of 370-555 MBq (10–15 mCi) of ^18^F-NaF. Scans were acquired in 3D mode, covering the body from the vertex to the feet, typically involving 12-14 bed positions with an acquisition time of 120 seconds per bed position. A non-contrast-enhanced low-dose CT scan was acquired for attenuation correction and anatomical localization using the following parameters: 140 kV, 80 mA, pitch of 1.375, and slice thickness of 3.75 mm.

***Image Reconstruction***

 Image reconstruction was performed using a three-dimensional ordered-subsets expectation maximization (3D OSEM) algorithm combined with time-of-flight (TOF) and point spread function (PSF) modeling, implemented commercially by GE Healthcare under the name SharpIR. The reconstruction parameters were set according to the manufacturer’s recommendations and are routinely applied in clinical practice as follows: Matrix size: 192×192, Iterations: 2, Subsets: 18 and Post-filter: Gaussian filter with a full-width at half maximum (FWHM) of 6.4 mm.

***Image Interpretation and Lesion Classification***

^18^F-NaF PET/CT images were reviewed on a dedicated GE Advantage Workstation. Two experienced nuclear medicine physicians, blinded to the final outcome initially, evaluated the scans. Axial, coronal, and sagittal planes of both the CT and fused PET/CT images were examined for lesion localization and characterization. All enrolled patients had at least one focus of positive skeletal ^18^F-NaF uptake. Each focal uptake lesion identified was subsequently classified as either true positive (TP) for metastasis or false positive (FP, representing benign uptake) based on a consensus review incorporating the PET/CT findings, clinicolaboratory data, findings from other imaging modalities (like MRI when available), histopathological results (if obtained), and follow-up imaging or clinical course. The specific criteria used for classification varied slightly based on the number of lesions described in Table 1.

**Table 1:** Represents the specific criteria based on the number of lesions used for classification of ^18^F NaF avid osseous lesions into true and false positive

**Number of lesions**	**Classification**	
	**True Positive**	**False Positive**
Solitary Lesions:	Focal ^18^F-NaF uptake corresponding to a CT-detected destructive lesion (with or without a soft tissue component), especially if associated with positive pathology, elevated tumor markers, or demonstrating sclerotic transformation on follow-up imaging. CT-detected sclerotic, subtle lytic, or marrow-based lesions with uptake were confirmed via local MRI or follow-up showing progression or sclerotic changes. Uptake corresponding to clear CT findings of arthritic/degenerative changes, fractures (especially with a history of trauma), or other benign processes without suspicious underlying lesions.	
Oligo (≤5) Lesions:	Uptake in lesions appearing purely lytic (with/without soft tissue), mixed lytic/sclerotic, especially with high clinical suspicion, confirmed by follow-up showing changes in number, size, or sclerotic transformation. For subtle CT lesions or suspected marrow-based lesions, confirmation via MRI at the most active site was sought. Uptake corresponding to CT-identified arthritic/ degenerative changes or lesions not meeting the TP criteria.	
Poly (>5) Lesions:	Uptake corresponding to multiple CT-detected lytic (with/without soft tissue), sclerotic, or mixed lesions, confirmed by follow-up. Uptake predominantly corresponding to CT-evident arthritic/degenerative changes and not fulfilling TP criteria.	

***Image Processing and Radiomics Feature Extraction***

**Figure 2:**
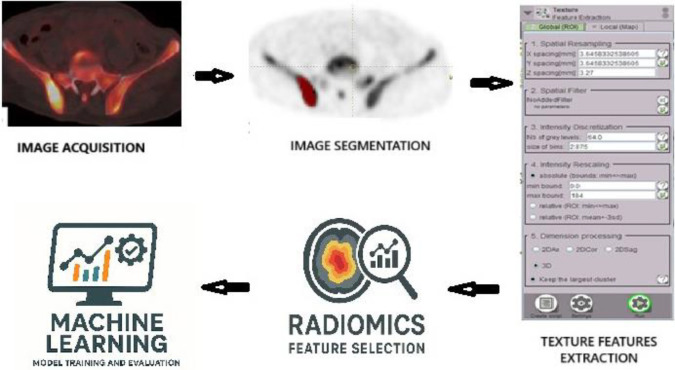
Overview of Radiomics Analysis workflow

 The LIFEx software package (version 7.7.0, www.lifexsoft.org) (13) was utilized for lesion segmentation and radiomic feature extraction. For each patient, the most active (highest uptake) bone lesion identified during the interpretation phase was selected for analysis. A region of interest (ROI) was manually delineated around the contour of this target lesion on the PET images by one of the experienced nuclear medicine physicians. Figure 2. No spatial resampling of the PET images was performed. Absolute intensity resampling was performed using a bin width of 2.8, i.e., 64 bins between 0 and 184 SUV. From each ROI, a total of 181 quantitative radiomic features were extracted from the ^18^F-NaF PET data. These included:

 First-order features (n=111): Morphological features (describing shape and size), conventional intensity-based features (e.g., SUV_max_, SUV_mean_, SUV_peak_), local intensity features, and features derived from the intensity histogram (e.g., skewness, kurtosis).

 Second-order textural features (n=70): Features quantifying spatial heterogeneity, including those derived from the Gray-Level Co-occurrence Matrix (GLCM), Gray-Level Run Length Matrix (GLRLM), Neighborhood Gray-Tone Difference Matrix (NGTDM), and Gray-Level Size Zone Matrix (GLSZM), as well as intensity based run-length matrix (RIM) features.

***Feature Selection***

 Feature selection was performed using Python with the Scikit-learn library (14) to enhance model efficiency, reduce dimensionality, and mitigate the risk of overfitting. A two-step process was employed on the training dataset (n=41):

1. Low-Variance Feature Removal: Features exhibiting very low variance across the training samples (variance threshold=0.01) were removed. This step eliminates near-constant features that provide little discriminatory information.

2. Univariate Feature Selection: The Analysis of Variance (ANOVA) F-test was applied to assess the statistical relevance of the remaining features concerning the target variable (TP vs. FP). The top 50 features with the highest F-scores (indicating the strongest individual association with the outcome) were selected for subsequent model building. This resulted in a final feature set of 50 radiomic parameters used for training and validating the machine learning models.

***Machine Learning Model Training and Evaluation***

***Initial Model Training***

 Three distinct machine learning classifiers were initially trained to evaluate the predictive capability of the selected radiomic features:

Support Vector Machine (SVM): Using a Radial Basis Function (RBF) kernel to capture potential non-linear relationships between features and the classification outcome.

 Random Forest (RF): An ensemble method consisting of 50 decision trees, with a maximum depth of 3 per tree.

 XGBoost (Extreme Gradient Boosting): A gradient boosting algorithm, also configured with 50 trees and a maximum depth of 3. Each model was trained using the 50 selected features from the entire training group (n=41) and subsequently evaluated on the independent validation group (n=21) to assess generalization performance.

***Cross-Validation and Model Refinement***

 To obtain more robust performance estimates and reduce potential bias from the single train/validation split, the training and validation datasets were combined (total n=62). A 5-fold cross-validation strategy was then applied to this combined dataset during a model refinement phase.

***Further enhancements were introduced during this phase***

 Recursive Feature Elimination (RFE): RFE, guided by an XGBoost estimator, was used to select the top 30 most important features from the initial 50, aiming for a more parsimonious and potentially more robust feature set.

 Polynomial Feature Expansion (SVM only): To potentially improve the SVM’s ability to model complex interactions, polynomial features (degree 2, interaction only terms assumed) were generated from the RFE-selected features specifically for the SVM model.

 Feature Scaling: MinMax scaling (scaling features to a [0, 1] range) was tested as an alternative to standardization (mean=0, std= 1), particularly for the tree-based models (RF and XGBoost), to potentially improve convergence or split quality.

 Hyperparameter Tuning: Hyperparameters for each model were fine-tuned based on performance within the cross-validation framework. The optimized parameters reported were:

– SVM: Kernel changed from RBF to Polynomial, C (regularization parameter) increased from 1 to 10, Gamma set to ’auto’.

– Random Forest: Number of trees increased from 50 to 100, max depth increased from 3 to 5, minimum samples required to split a node increased from 2 to 4.

– XGBoost: Number of trees increased from 50 to 150, max depth increased from 3 to 4, learning rate decreased from 0.1 to 0.05, L1 regularization (alpha) added (0.1).

***Statistical Analysis***

 Descriptive statistics were used to summarize patient characteristics. Continuous variables (like age, SUV metrics) were reported as mean standard deviation or median (range), while categorical variables (like sex, primary site, lesion classification) were reported as counts and percentages. Comparisons between quantitative variables were done using the non-parametric Kruskal-Wallis and Mann-Whitney tests (15). A *P*-value<0.05 was considered statistically significant. Comprehensive machine learning–based statistical models were applied to analyze the radiomics features. Specifically, three classifiers were evaluated: Support Vector Machine (SVM), Random Forest, and Extreme Gradient Boosting (XGBoost). These models were trained on a training dataset (n=41) and validated on an independent dataset (n=21). To ensure robustness, we further applied 5-fold cross-validation with recursive feature elimination (RFE) and hyperparameter tuning. Model performance was evaluated using several metrics derived from the confusion matrix, calculated for both the training/cross-validation phase and the independent validation set. Key metrics included: Accuracy, Specificity, Sensitivity, and the Area Under the Receiver Operating Characteristic Curve (AUC). The AUC provides a summary measure of the model’s ability to discriminate between the TP and FP classes across all possible classification thresholds. Statistical analyses were performed using Python (Scikit-learn) and the statistical package for the Social Sciences (SPSS) version 28 (IBM Corp., Armonk, NY, USA).

## Results

***Patient Characteristics***

 The study cohort comprised 62 patients in total, randomly split into a training group (n=41) and a validation group (n=21). The characteristics of the training group (n=41) are summarized in Table 1. The median age was 53 years (range: 30-83 years). The group consisted of 12 males (29.3%) and 29 females (70.7%). The primary malignancies varied, with breast cancer being the most common (n=26, 63.4%), followed by prostate cancer (n=4, 9.8%), lung cancer (n=4, 9.8%), mesothelioma (n=2, 4.9%), hepatocellular carcinoma (HCC) (n=2, 4.9%), malignancy of unknown origin (MUO) (n=2, 4.9%), and pancreatic cancer (n=1, 2.4%). All patients were referred for ^18^F-NaF PET/CT for 

initial staging or assessment of suspected bone metastases. Based on the consensus interpretation incorporating clinic-laboratory and follow-up data, 17 patients (41.5%) in the training group were classified as having true positive (TP) metastatic lesions, while 24 patients (58.5%) were classified as having false positive (FP) benign uptake. Table 2.

**Table 2:** Clinical characteristics of the enrolled training group (n=41)

**Characteristic**	**Category**	**Count**	**Percentage (%)**
**Sex**	Male	12	29.3
	Female	29	70.7
**Primary Site**	Breast	26	63.4
	Prostate	4	9.8
	Lung	4	9.8
	Mesothelioma	2	4.9
	HCC	2	4.9
	MUO	2	4.9
	Pancreas	1	2.4
**Number of Lesions**	Solitary	10	24.4
	Few (Oligo, <5)	19	46.3
	Multiple (Poly, >5)	12	29.3
**Lesions Site(s)**	Axial	25	61.0
	Appendicular	2	4.8
	Axial and Appendicular	14	34.1
**Final Diagnosis (TP)**	Lytic Metastases	6	14.6
	Mixed Metastases	7	17.1
	Sclerotic Metastases	4	9.8
**Final Diagnosis (FP)**	Degenerative	12	29.3
	Arthritic	10	24.4
	Traumatic	1	2.4
	Infection	1	2.4
**Lesion Classification**	True Positive (TP)	17	41.5
False Positive (FP)	24	58.5

HCC: Hepatocellular Carcinoma; MUO: Metastases of Unknown Origin

***Comparison of SUV Metrics***

 Standard uptake values (SUV) derived from the ^18^F-NaF PET scans were compared between the TP and FP groups within the training cohort (Table 2). Significantly higher SUV_max_ and SUV_mean_ values were observed in patients with TP lesions compared to those with FP lesions. 

The mean (±SD) SUV_max_ was 61.34 (±41.46) in the TP group versus 29.09 (±12.80) in the FP group (p=0.006). Similarly, the mean (±SD) SUV_mean_ was 24.51 (±15.46) in the TP group versus 14.94(±6.00) in the FP group (*p* = 0.034). Median values also showed a clear difference (SUV_max_: 43.7 vs 28.55; SUV_mean_: 21.5vs14.3) (Table 3).

**Table 3:** Comparison of ^18^F-NaF PET SUV metrics between True Positive (TP) and False Positive (FP) lesions in the training group (n=41)

**Metric**	**TP Group (n=17) Mean±SD (Median)**	**FP Group (n=24) Mean±SD (Median)**	***P*** **-value**
SUV_max_	61.34 ± [41.46] (43.7)	29.09 ± [12.80] (28.55)	0.006
SUVmean	25.51 ± [15.46] (21.5)	14.94 ± [6.00] (14.3)	0.034

**Figure 3:**
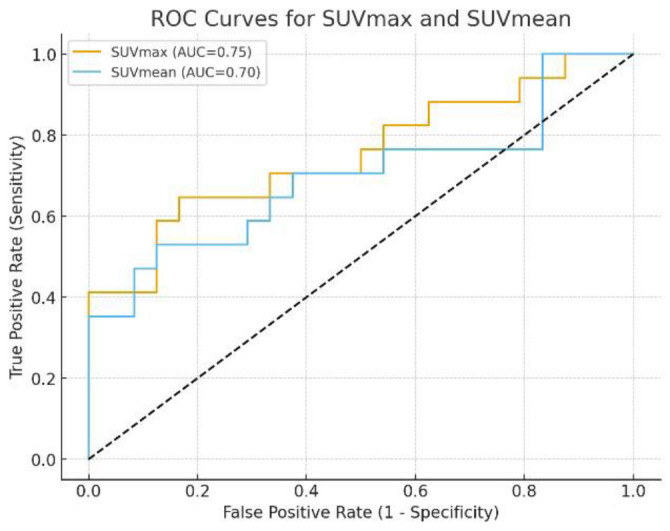
Receiver Operating Characteristic (ROC) curves for SUV_max_ and SUV_mean_ that discriminate TP from FP bone lesions

 ROC curve analysis was performed using SUV_max_ and SUV_mean_ values from the provided dataset (n=41) to mark cutoff points that discriminate TP from FP bone lesions. ROC curve marked an optimal cutoff (Youden Index): 37.7, with sensitivity of 64.7%, Specificity of 83.3%, Positive Predictive Value (PPV)= 73.3%, Negative Predictive Value (NPV) of 76.9%, Accuracy of 75.6% and AUC=0.75 (*P*-value=0.006). An Optimal cutoff (Youden Index): ≥17.6 was marked for SUV_mean_ with Sensitivity of 70.6%, Specificity of 70.8%, PPV of 63.2%, NPV of 77.3%, Accuracy=70.7% and AUC=0.70 (*P*-value=0.034) (Figure 3).

***Machine Learning Model Performance***

***Initial Validation Performance***

 The initial performance of the three machine learning models (SVM, Random Forest, XGBoost), trained on the 41 training samples and tested on the 21 validation samples using the 50 ANOVA-selected features, is presented in Table 3. The XGBoost model demonstrated the highest performance on the training set (Accuracy: 97.6%, Specificity: 92.3%, Sensitivity: 88.9%, AUC: 0.98 (95%CI: 0.94–1.00)). On the independent validation set, XGBoost also achieved the best results among the three models, with an accuracy of 76.2%, specificity of 75.0%, sensitivity of 60.0%, and an AUC of 0.78 (95%CI: 0.61–0.91). Random Forest showed good training performance (Accuracy: 92.7%, AUC: 0.94 (95%CI: 0.88-0.98)) but lower validation performance (Accuracy: 71.4%, AUC: 

0.72 (95%CI: 0.54–0.86)), suggesting some overfitting. The SVM model exhibited the weakest generalization (Validation Accuracy: 66.7%, AUC: 0.65 (95%CI: 0.47–0.81)) Table 4.

**Table 4:** Initial diagnostic performance of machine learning models on training (n=41) and validation (n=21) sets using 50 features

**Model**		**Accuracy (%)**		**Specificity (%)**		**Sensitivity (%)**		**AUC**	
		**Train**	**Valid**	**Train**	**Valid**	**Train**	**Valid**	**Train**	**Valid**
SVM		85.4	66.7	80.0	50.0	75.0	40.0	0.87 (95%CI: 0.78–0.93)	0.65 (95%CI: 0.47–0.81)
Random Forest		92.7	71.4	85.7	66.7	80.0	50.0	0.94 (95% CI: 0.88–0.98)	0.72 (95%CI: 0.54–0.86)
XGBoost		97.6	76.2	92.3	75.0	88.9	60.0	0.98 (95% CI: 0.94–1.00)	0.78 (95%CI: 0.61–0.91)

SVM: Support Vector Machine; AUC: Area Under the Receiver Operating Characteristic Curve. 95%CI: 95% Confidence Interval

***Cross-Validation Performance***

 Using the combined dataset (n=62) and 5-fold cross-validation with the initial 50 features, the performance estimates became more robust (Table 4). XGBoost maintained its superiority with a mean accuracy of 80.1% (±3.5%) and a mean AUC of 0.82 (95%CI: 0.75–0.88). Random Forest achieved a mean accuracy of 76.8% (±3.9%) and a mean AUC of 0.76 (95%CI: 0.68-0.83)). SVM performance improved slightly compared to the single validation split but remained the lowest (Mean Accuracy: 73.5% ± 4.2%, Mean AUC: 0.70 (95% CI: 0.61–0.78)) (Table 5).

**Table 5:** Performance metrics of machine learning models with 5-fold cross validation on the combined dataset (n=62) using 50 features

Model	Accuracy (Mean ±Std)	Specificity (Mean %)	Sensitivity (Mean %)	F1 Score (Mean %)	AUC-ROC (Mean) (95%CI)
SVM	73.5% ± 4.2%	61.2%	55.8%	58.3%	0.70 (95%CI: 0.61–0.78)
Random Forest	76.8% ± 3.9%	68.9%	60.2%	64.3%	0.76 (95%CI: 0.68–0.83)
XGBoost	80.1% ± 3.5%	74.5%	66.7%	70.4%	0.82 (95% CI: 0.75–0.88)

Performance metrics reported as mean percentage across 5 folds, except for Accuracy (Mean±StandardDeviation) and AUC-ROC (mean) (95% confidence interval)

***Performance after Feature Engineering and Tuning***

**Figure 4:**
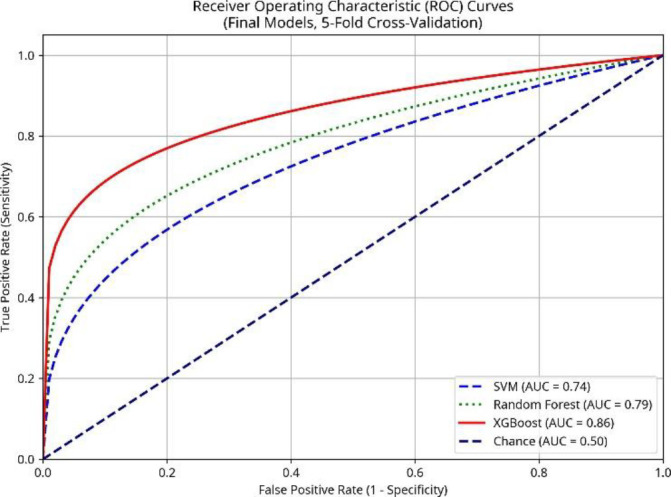
Receiver Operating Characteristic (ROC) curves for the final optimized machine learning models (SVM, Random Forest, XGBoost) evaluated using 5-fold cross-validation on the combined dataset (n=62). The Area Under the Curve (AUC) is reported for each model

 Significant improvements were observed after applying RFE (reducing to 30 features), specific feature engineering steps (polynomial features for SVM, Min Max scaling for tree models), and hyperparameter tuning, evaluated again using 5-fold cross-validation on the combined dataset (Table 6). The optimized XGBoost model achieved the highest performance with a mean accuracy of 85.7% (±2.9%), precision of 81.4%, recall (sensitivity) of 74.2%, F1−score of 77.6%, and an AUC of 0.86 (95% CI: 0.78–0.92). The tuned Random Forest model also showed strong performance (Mean Accuracy: 80.5% ± 3.2%, AUC: 0.79 (95% CI: 0.70–0.87)). The SVM model benefited notably from the polynomial features and tuning, reaching a mean accuracy of 78.2%± 3.7% and an AUC of 0.74 (95% CI: 0.63 – 0.82) (Figure 4, Table 6).

**Table 6:** Final performance metrics of machine learning models after feature engineering and hyperparameter tuning, evaluated with 5-fold cross-validation (n=62) using 30 RFE- selected features

**Model**	**Accuracy** **(K-Fold Mean±Std)**	**Precision** **(Mean %)**	**Recall** **(Mean %)**	**F1 Score** **(Mean %)**	**AUC-ROC (Mean)** **(95%CI)**
SVM	78.2% ± 3.7	66.8%	63.5%	65.1%	0.74 (95% CI: 0.63 – 0.82)
Random Forest	80.5% ± 3.2	72.1%	67.3%	69.6%	0.79 (95% CI: 0.70 – 0.87)
XGBoost	85.7% ± 2.9	81.4%	74.2%	77.6%	0.86 (95% CI: 0.78 – 0.92)

Performance metrics reported as mean percentage across 5 folds, except for Accuracy (Mean±StandardDeviation) and AUC-ROC (mean) (95% confidence interval)

***Comparative Analysis: SUV Metrics vs Machine Learning***

 Head-to-Head Comparison (DeLong's Test for Correlated ROC Curves) was used to compare XGBoost vs SUV metrics and revealed XGBoost radiomics model significantly outperformed SUV_max_ (AUC=0.86 vs 0.75, p=0.002) and SUV_mean_ (AUC=0.86 vs 0.70, p<0.001) (Table 7).

**Table 7:** Head-to-Head Comparison (DeLong's Test for Correlated ROC Curves)

**Comparison**	**AUC Difference**	**95% CI**	***P*** **-value**
XGBoost vs SUV_max_	0.11	0.04–0.18	0.002
XGBoost vs SUV_mean_	0.16	0.08–0.24	<0.001
XGBoost vs Combined SUV	0.09	0.02–0.16	0.011

***Feature Importance***

**Figure 5:**
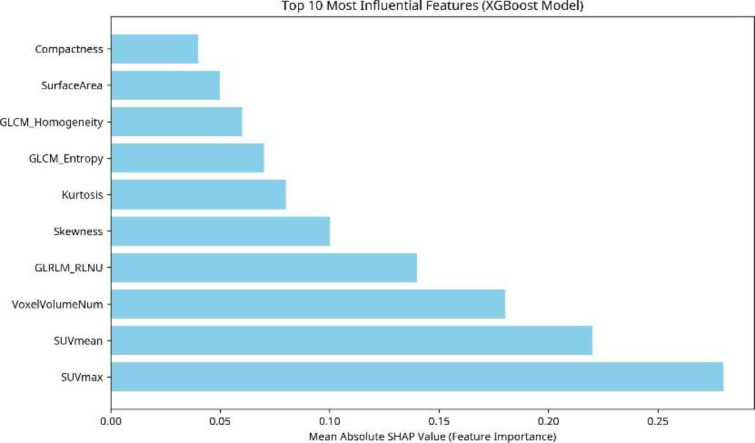
Top 10 most influential features identified by SHAP analysis for the final optimized XGBoost model. Feature importance is quantified by the mean absolute SHAP value across the cross-validation folds

 Feature importance for the final, optimized XGBoost model was assessed using SHAP (SHapley Additive Explanations) values to understand the contribution of individual radiomic features to the models’ predictions. The top 10 most influential features are listed in Table 6. Conventional PET metrics, SUV_max_ and SUV_mean_, emerged as the most dominant predictors. Features related to tumor volume, shape (Surface Area, Compactness), and texture/heterogeneity (Heterogeneity Index, Skewness, Kurtosis, Entropy, Homogeneity) also contributed significantly to the models ability to discriminate between true positive and false positive lesions (Figure 5).

## Discussion

**Figure 6:**
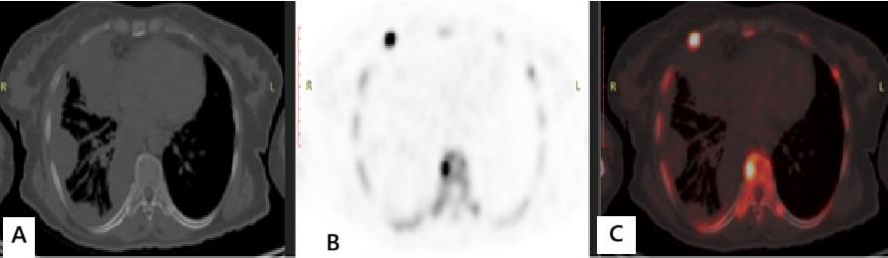
57-years-old female with primary mesothelioma. Image (**A**): CT bone window free from any sclerotic or lytic lesions. Images (**B** and **C**) PET and fused PET/CT images revealed dorsal vertebral and right rib ^18^F NaF avid metastatic bone lesions. This case illustrates the superiority of functional imaging over anatomical imaging alone

 Recent technological advancements in PET/CT imaging and challenges related to the supply of ^99m^Tc have led to a renewed interest in ^18^F-NaF PET/CT for the detection and assessment of bone metastases (4, 2). ^18^F-NaF offers favorable pharmacokinetics, including faster blood clearance and higher bone uptake compared to ^99m^Tc-MDP, which, combined with the superior spatial resolution of PET, potentially allows for improved lesion detection compared to morphological imaging alone (3) (Figure 6). However, a significant limitation of ^18^F-NaF PET/CT is its suboptimal specificity. 

 Fluoride ion accumulation reflects osteoblastic activity and bone turnover, which is not exclusive to metastatic processes. Increased uptake can be observed in various benign conditions, such as fractures, degenerative joint disease, arthritis, and inflammatory processes (4, 16). This challenge is particularly pronounced in elderly patients, who often have a higher prevalence of both metastatic disease and benign degenerative bone conditions, leading to overlapping uptake patterns and potential false-positive interpretations (16). 

 While the addition of CT in hybrid PET/CT imaging aids in anatomical correlation and characterization of underlying bone structure, differentiating benign from malignant uptake can remain difficult, resulting in equivocal findings and inconsistent diagnostic performance across different studies and primary tumor types (17, 18). Previous studies evaluating ^18^F-NaF PET/CT for bone metastasis detection have reported variable diagnostic accuracy. For instance, Withofs et al. (17) reported sensitivity, specificity, and accuracy of 76, 84.2, and 80%, respectively. 

 Radiomics, the extraction of high-dimensional quantitative data from medical images, offers a potential avenue to improve the characterization of lesions beyond visual assessment or simple SUV metrics (6, 7). By applying machine learning algorithms to these rich feature sets, radiomics aims to build predictive models that can enhance diagnostic accuracy and support clinical decision-making (9).

 In this study, we explored the utility of ^18^F-NaF PET radiomics combined with machine learning to differentiate true metastatic lesions from false-positive benign uptake. We evaluated three common classifiers: SVM, Random Forest, and XGBoost. Our initial results, using a simple train/validation split, indicated that XGBoost provided the best performance, achieving a validation AUC of 0.78, superior to Random Forest (AUC: 0.72) and SVM (AUC: 0.65). This aligns with findings from other radiomics studies where gradient boosting methods often perform well. For example, Wang et al. (19) used XGBoost for feature selection combined with a KNN classifier to detect early bone metastases on CT, achieving high AUCs. Similarly, Zhou et al. (10), in the context of ^18^F-FDG PET/CT for lung lesions, identified GBDT (a related gradient boosting method) and Random Forest as optimal classifiers. However, our initial SVM model performed poorly, particularly in terms of sensitivity on the validation set. Recognizing the limitations of a single train/validation split and the potential for model improvement, we implemented a more robust evaluation using 5-fold cross-validation and incorporated advanced feature engineering and hyperparameter tuning. This iterative refinement process led to substantial performance gains across all models. The final optimized XGBoost model achieved a mean AUC of 0.86 and accuracy of 85.7%.

 Our analysis of feature importance using SHAP values revealed that traditional SUV metrics (SUV_max_, SUV_mean_) were the most influential predictors, consistent with clinical practice and previous findings showing higher uptake in metastatic lesions compared to most benign processes (20). Our data confirmed significantly higher SUV_max_ and SUV_mean_ in TP lesions (p=0.006 and p=0.034, respectively). However, the SHAP analysis also highlighted the added value of radiomic features. Morphological features (Volume, Surface Area, Compactness) and textural/heterogeneity features (GLRLM RLNU, Skewness, Kurtosis, GLCM Entropy, GLCM Homogeneity) ranked among the top 10 predictors, suggesting that quantitative assessment of lesion size, shape, and internal uptake patterns provides complementary information beyond simple intensity metrics, contributing to the improved discriminatory power of the machine learning models. Also, ROC curve analysis was performed using SUV_max_ and SUV_mean_ values to mark cutoff points that discriminate TP from FP bone lesions. ROC curve marked an optimal SUV_max_ cutoff point: ≥37.7, with accuracy of 75.6% and AUC=0.75 and an optimal SUV_mean_ cutoff: ≥17.6 with accuracy of 70.7% and AUC of 0.70. Head-to-Head Comparison was also used to compare XGBoost vs SUV metrics and revealed XGBoost radiomics model significantly outperformed SUV_max_ (AUC=0.86 vs 0.75, p=0.002) and SUV_mean_ (AUC=0.86 vs 0.70, p<0.001). These findings indicate moderate discriminative ability for SUV metrics alone, underscoring the added value of radiomics and ML models.

 This study has several limitations. Firstly, the sample size (n=62) is relatively small for machine learning applications, which can limit the generalizability of the findings and increase the risk of overfitting, although cross-validation and feature selection methods were employed to mitigate this. Secondly, the study is retrospective and conducted at a single institution, potentially introducing selection bias. The reference standard for classifying lesions relied on a combination of imaging, clinical data, and follow-up, rather than histopathology for all lesions, which is the gold standard but often impractical. Thirdly, while we explored several feature engineering and tuning steps, the optimal pipeline might require further investigation. Also, there is narrow distribution of benign etiologies in current study that could artificially inflate the apparent specificity of the ML models. Thus, further testing the model on broader set of benign etiologies is recommended so the diagnostic performance will better fully represent its real-world utility. The analyses were performed only on per patient basis not per lesion. Finally, the lack of an independent external validation cohort prevents a definitive assessment of the model’s performance in different patient populations or acquisition settings. Future work should focus on validating these findings in larger, multi-center cohorts with standardized protocols and ideally incorporating histopathological confirmation where possible. 

## Conclusion

 This study demonstrates that radiomic features extracted from ^18^F-NaF PET images, when analyzed using machine learning classifiers, can aid in distinguishing true metastatic bone lesions from false-positive benign uptake. The application of radiomics based models, particularly the optimized XGBoost classifier developed through rigorous feature selection and hyperparameter tuning, showed promising diagnostic performance, achieving a mean accuracy of 85.7%.
